# Fully robotic social environment for teaching and practicing affective interaction: Case of teaching emotion recognition skills to children with autism spectrum disorder, a pilot study

**DOI:** 10.3389/frobt.2023.1088582

**Published:** 2023-05-03

**Authors:** Pegah Soleiman, Hadi Moradi, Bijan Mehralizadeh, Hamed Ameri, Rosa I. Arriaga, Hamid Reza Pouretemad, Negin Baghbanzadeh, Leila Kashani Vahid

**Affiliations:** ^1^ School of ECE, University of Tehran, Tehran, Iran; ^2^ Intelligent Systems Research Institute, Sungkyunkwan University, Suwon, Republic of Korea; ^3^ School of Mechanical Engineering, University of Tehran, Tehran, Iran; ^4^ Department of Psychology, University of Tehran, Tehran, Iran; ^5^ School of Interactive Computing, Georgia Institute of Technology, Atlanta, GA, United States; ^6^ Department of Psychology, Shahid Beheshti University, Tehran, Iran; ^7^ Department of Psychology, Islamic Azad University Science and Research Branch, Tehran, Iran

**Keywords:** robotic social environment, social interaction, observational learning, emotion recognition, autism

## Abstract

21st century brought along a considerable decrease in social interactions, due to the newly emerged lifestyle around the world, which became more noticeable recently of the COVID-19 pandemic. On the other hand, children with autism spectrum disorder have further complications regarding their social interactions with other humans. In this paper, a fully Robotic Social Environment (RSE), designed to simulate the needed social environment for children, especially those with autism is described. An RSE can be used to simulate many social situations, such as affective interpersonal interactions, in which observational learning can take place. In order to investigate the effectiveness of the proposed RSE, it has been tested on a group of children with autism, who had difficulties in emotion recognition, which in turn, can influence social interaction. An A-B-A single case study was designed to show how RSE can help children with autism recognize four basic facial expressions, i.e., happiness, sadness, anger, and fear, through observing the social interactions of two robots speaking about these facial expressions. The results showed that the emotion recognition skills of the participating children were improved. Furthermore, the results showed that the children could maintain and generalize their emotion recognition skills after the intervention period. In conclusion, the study shows that the proposed RSE, along with other rehabilitation methods, can be effective in improving the emotion recognition skills of children with autism and preparing them to enter human social environments.

## 1 Introduction

Humans are social agents (Baumeister & Leary, 1995), so they tend to have a social life by belonging to different groups of people in a society. This social life makes it crucial for people to learn from others. What humans learn through their social lives is called social learning, which might happen directly through training and/or, indirectly through observing others’ behaviors. In fact, an important part of social learning occurs through observational learning (Bandura, & McDonald, 1963; Bandura, 1965) which refers to a learning process of acquiring new responses by observing other people’s behaviors and the contingencies of those behaviors. Observational learning is considered an important skill that starts to develop in childhood **(** Bandura, 1963 **)**. With observational learning ability, we can learn both personal (Taylor et al., 2012), social, and interpersonal behaviors (Shukla-Mehta et al., 2010). Therefore, a social environment provides us with a lot of learning opportunities.

Typically Developing (TD) children can acquire many social skills in their natural environment from the beginning of their life when they observe their parents and relatives. What children experience and observe in their environment can provide them with incredible experiences for learning, which might be crucial for their lifelong physical and mental health (O’Connell et al., 2009). In the past decade, the widespread availability of smartphones and tablets has increased concerns about too much screen time for children. Unfortunately, the increase in screen time has been associated with an increase in autistic behaviors (Chen et al., 2021). Furthermore, it has been shown that the increase in screen time is related to a decrease in social skills, especially in children with no siblings (Hu et al., 2020). The above findings combined with the fact that children with autism have difficulty participating in social environments or get rejected from their peer groups or social environments (Sari et al., 2021), highlight the importance of providing environments for children with autism to practice more interactions and to improve their social skills.

To be more specific, children with autism show considerable difficulties in communication, social skills, and repetitive or stereotypic behaviors based on the Diagnostic and Statistical Manual of Mental Disorders (American Psychiatric Association, 2013). They have limited or delayed social abilities so it is likely that they show difficulty in the required skills for successful observational learning, such as imitation (Rogers & Williams, 2006), and consequence discrimination (Pereira & Greer, 2009). Consequently, they may have problem learning by observation. It has been documented that children with autism are less likely to learn through observation compared to TD children (Taylor et al., 2012). So, despite all efforts to improve their skills in 1-to-1 therapy formats, they may be deprived of learning in social environments and lose the chance of coping with new and/or complex social situations (Chan et al., 2009; Vander Wiele, 2011). In addition, 1-to-1 therapies need the availability of staff with extensive training and resources to provide therapy for every single child with special needs in most countries (Iadarola et al., 2015).

Consequently, there have been methods to, explicitly or implicitly, compensate for the declined/eliminated social learning opportunities. These methods can be categorized into 1) human-based group instruction therapies (Urlacher et al., 2016), 2) peer-mediated interventions (Kamps et al., 2017), and 3) video modeling (Alkinj et al., 2022). Unlike 1-to-1 therapies, these methods can provide resources for multiple children simultaneously (Collins et al., 1991). Although video modeling is more repeatable and can be designed with virtual characters (Sherrow et al., 2015), group instruction and peer-mediated methods provide more opportunities to practice social skills in an environment that is close to typical classrooms (Hume & Campbell, 2019). Children can learn social skills such as empathy (Leaf et al., 2010), appropriate group play manners, making conversations among a group (Barry et al., 2003), and practicing their acquired knowledge in a more naturalistic environment, which could lead to giving them a chance of generalization of those skills (Watkins et al., 2015). However, these methods still require skilled human intervention, which in turn requires therapists and trained peers to model the target behaviors reliably during therapy sessions (DiSalvo & Oswald, 2002). In addition, managing sessions and maintaining children’s attention are more difficult than 1-to-1 therapies and may need additional support (Colozzi et al., 2008). Moreover, the time needed for preparing group therapy sessions makes them less preferred by teachers (Kamps et al., 1992). Finally, when the target skills involve more than one person to model interpersonal interactions, it becomes more challenging than 1-to-1 therapies or even simple group therapies. Thus, social learning therapies need a team of teachers and students in order to be able to demonstrate target behaviors.

Therefore, different assistive technology tools, such as educational mobile applications (Marin et al., 2019), computer games (Valencia et al., 2019), and robots (Boccanfuso et al., 2017) have been recently designed to use in the therapy and education of children with autism. They can simulate social environments, which are predictable, controllable, repeatable, and less complex than human social environments (Good et al., 2016). Also, it has been shown that they can be attractive and engaging for children with autism (Scassellati et al., 2012; Mavadati et al., 2016) which in turn may lead to gaining better outcomes from therapies. In addition, among these assistive technologies, the embodiment of social robots makes them suitable to model human-human interaction in a physical setup (Scassellati et al., 2012). Furthermore, robots allow touch and tactile exploration, which makes the education multi-channel, and in turn, increases the level of efficacy of education and companionship (Burns et al., 2020). Researchers have tried to use robots in different therapies, in which they have shown that social robots could help to improve a wide range of children’s abilities (Henschel et al., 2021), such as imitation (Zheng et al., 2015), joint attention (Anzalone et al., 2014), turn taking (Soleiman et al., 2016; Boccanfuso et al., 2017), emotion recognition and regulation (Azuar et al., 2019; Rocha et al., 2022), and social and academic skills (Qidwai et al., 2019). It should be noted that the control of these robots, ranges from simple remote-control robots (Scassellati et al., 2012) to semi/fully autonomous robots (Melo et al., 2019; Wood et al., 2021) to show the efficacy of social robots in clinical or at-home setups (Scassellati et al., 2018). In addition, there are studies that have tried to compare robot-based therapies with human-based therapies (Ghiglino et al., 2021). Holeva et al. (2022) addressed socioemotional, cognitive, and behavioral issues related to ASD. They compared an intervention with NAO robot to an intervention by humans only. The results indicated significant clinical improvements in both interventions. However, parents, teachers, and children were more satisfied with the robot-based intervention compared to the human-only intervention. According to these previous studies and several reviews and survey papers (Alabdulkareem et al., 2022; Kouroupa et al., 2022), it has been concluded that robotic-based therapy is a promising field and can motivate and engage children to participate in different activities (Conti et al., 2020; Rakhymbayeva et al., 2021). However, there are mixed results of clinical effectiveness (Duquette et al., 2008; Tapus et al., 2012) in some cases and the research is still insufficient and needs more investigations to address the challenges about the efficacy and effectiveness of using robots in ASD therapy.

In almost all the mentioned studies, the researchers used a single robot that has no potential to model a social interaction situation without human involvement. So, the following two challenges can be addressed in single robot-based social environments: 1) a full social interaction may not happen since, in robot-therapist sessions, children with autism get more engaged with the robot than the therapist (Meucci, 2019; Kostrubiec, 2020), 2) the therapy process needs trained humans, which limits the usage of the method. Furthermore, it is not possible to model social and interpersonal skills such as turn-taking and empathy without the help of a human and benefit from observational learning. This was the reason that we proposed a fully Robotic Social Environment (Soleiman et al., 2020). Also, there are a few attempts to provide social environments without human involvement. For instance (Saadatzi et al., 2018), used a robot and a virtual human teacher to simulate a classroom in which the robot is a peer for children with autism. This study showed that the children could learn the sight words that were instructed to the robot by the virtual teacher through observational learning. Ali et al. (2019), used two NAO robots and claimed that using multi-robot can improve the multi-human communication skills of ASD children. Similarly, So et al. (2020) used two NAO robots and showed improvement in children’s joint attention and functional play behavior ability after watching the robots’ playing drama. However, there was no evidence to show better performance in the pretend play behavior of participating children.

In our proposed Robotic Social Environments (RSE) multiple robots are used to model both non-social and social behaviors. This approach can reduce the need for human involvement and assistance in behavior modeling. Such an environment: 1) can be used to practice and learn social skills, 2) can run without human involvement and eliminate the hesitation of children with autism to interact with humans, 3) making it available for infants from early ages that can reduce the impacts of autism, and 4) this approach has less dependency on the availability of human therapists. It should be noted that such an environment, as it is a simulation of a real social environment, can be a supplementary tool that might be able to prepare children to attend to human social environments.

In previous research, Soleiman et al. (2020) showed that children with autism could learn to play with a robotic ball after observing two parrot-like robots play with it. In addition, they could join the robots’ group play and take a turn. So, it suggested that this kind of environment has a good potential to simulate the human social environment and children can learn by observing and participating in this environment. Despite proving the capability of using an RSE for teaching non-social capabilities to children with ASD, that study did not show the capability of an RSE in teaching social skills. Thus, in the current study, a single-subject ABA pilot experiment was designed to see whether an RSE is beneficial to teach understanding and recognizing the emotions of other people to children through observational learning. In other words, we answered the following question: Is it possible to teach emotion recognition to children with autism using an RSE without human intervention? In this study, we chose four basic facial expressions, i.e., happiness, sadness, anger, and fear, that children with autism have difficulty with (Uljarevic & Hamilton, 2013). The approach was tested on six participants and the results showed significant improvement in their emotion recognition capability after 9 sessions.

It should be noted that we do not want to compare the efficacy and effectiveness of our approach to other approaches and studies for emotion recognition therapy using robots (Conti et al., 2019; Lebersfeld et al., 2019) or without using robots (Chen et al., 2016; Fridenson-Hayo et al., 2017; Petrovska & Trajkovski, 2019). Rather, we want to show that the proposed RSE can be used for emotion recognition therapy without help from a human therapist. In other words, the advantage of our proposed approach is its independence from human intervention in comparison to human-centered approaches. The advantage of the proposed approach to application-based approaches with no human intervention is in the embodiment of the robots and their attractiveness to children with ASD.

## 2 Related work on emotion recognition and expression using robots

The selection of emotion recognition social skills was because children with autism usually show difficulties in interpreting emotional cues in facial expressions (Keating & Cook, 2020). Impairment in this ability can negatively affect their social life, especially when they enter school, and it is needed for them to interpret the emotions of their peers. Therefore, treatments are necessary to prevent social isolation for these children. Toward this goal, there were several attempts to teach emotional expression or recognition to children with autism through facial expressions (Soares et al., 2019; Silva et al., 2021; Singh et al., 2023). In most of these studies, there were robots developed with facial expressions that might be expressed mechanically or via an LCD. On the other hand, there were robots that did not have a clear facial expressions (Mavadati et al., 2016; Conti et al., 2019; Nagae & Lee, 2022). Thus, in these robots, additional materials, such as real or cartoon-like images were used to teach facial expressions by giving explanations, instructions, or feedback to children. In addition, there were studies that focused on detecting the emotional and behavioral states of children with ASD for better affective interaction with ASD children (Rudovic et al., 2018; Kim et al., 2021; Silva et al., 2021; Lytridis et al., 2022; Valagkouti et al., 2022). In these studies, different features, such as facial expressions, gestures, and eye gaze duration, were used to measure the engagement and affective state of ASD children.

In all these studies single robots were used to conduct therapies. In other words, the use of multiple robots in a simulated social environment has not been studied before. That is why we investigated the usage of an RSE to teach such a social skill through observational learning.

## 3 Methods

### 3.1 The robots

**FIGURE 1:**
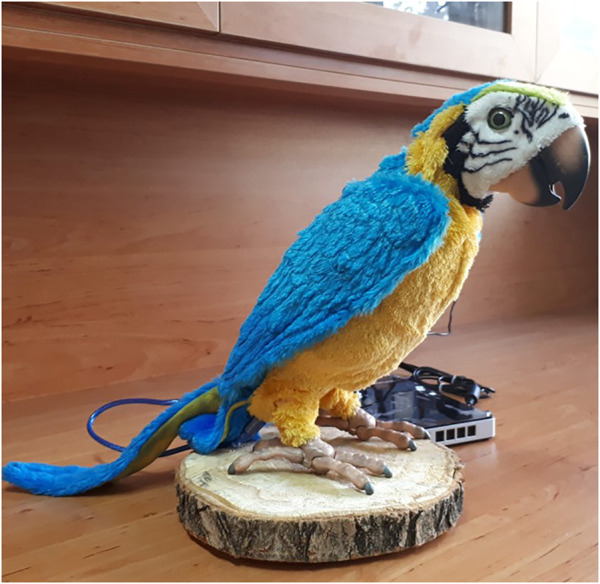
RoboParrot.

**FIGURE 2:**
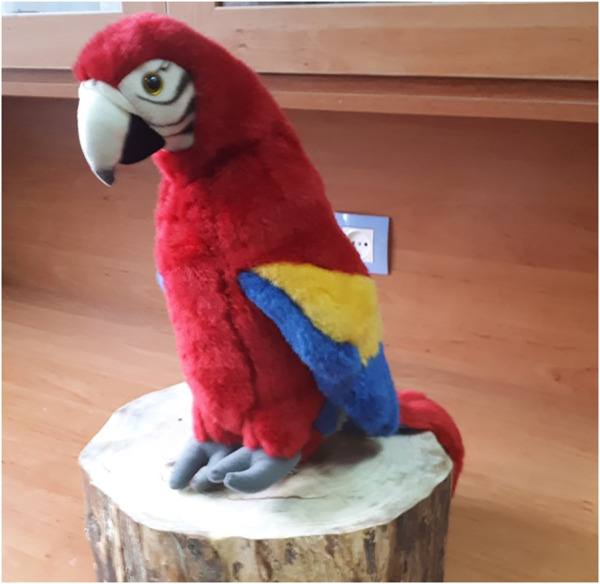
Red robot.

Two parrot-like robots were used in this experiment (Figures 1, 2). The first robot that has been developed in our laboratory is RoboParrot (Figure 1), a robot based on a toy from Hasbro Company. Two motors facilitate the robot’s body, mouth, and eye movements. The other robot (Figure 2) is called *Red*, a fluffy toy with a servo motor embedded under its wooden stand to enable it to have a pan movement. Both robots are equipped with speakers to be used for voice-based interaction. The voices of two adult women were changed to make them funny and childlike. When RoboParrot speaks, its mouth opens and closes. When *Red* speaks its body turns left and right.

In the previous version of the robots’ controlling systems (Soleiman et al., 2020), each robot was controlled by an independent system, so we needed two operators to control the robots. In the new version, the robots are controlled through a unified ROS platform, using the ROS multiple-machine framework. ROS makes it possible to have a high modularity and maintainability architecture and reduces the cost of system development, expansion, and customization. Using the distributed architecture of ROS multiple machines has great advantages such as fault resiliency that guarantees a more robust system. To remotely operate the robots, a web application was developed with two sections color-matched with each robot.

### 3.2 Participants

Regarding participants, all ethical standards were observed. All families of the participating children received a letter explaining the goals, experimental procedure, and the rights of parents and children. The parents signed a consent form in accordance with the Declaration of Helsinki.

The participants were six children, four with a diagnosis of severity level 1, requiring minimal support. One with a diagnosis of severity level 2, requiring substantial support, based on the Gilliam Autism Rating Scale (GARS) (Gilliam, 2014). One with a developmental delay diagnosis. In addition, the diagnosis of the participants was done, at least by a psychologist and a psychiatrist independently. The inclusion criteria for children were: 1) being able to express at least a two-word sentence independently to be able to express what they perceive from the sessions or answer the questions about the robots, 2) not having problems in their vision or hearing, 3) being present in an autism center, where the sessions were conducted, at least once a week for their normal therapies to reduce commuting due to coronavirus pandemic, 4) reported having no major disruptive behavior to reduce the probability of damaging robots. One female participant (P1) was introduced by a charity for autism and the other five male participants (P2, P3, P4, P5, P6) were recruited from a local autism center. The demographic characteristics of the participants can be seen in Table 1.

**TABLE 1:** The demographic characteristics of the participants.

Participant	Gender	Age	Disability
P1	Female	10	Autism
P2	Male	6	Autism
P3	Male	7	Developmental delay
P4	Male	8	Autism
P5	Male	11	Autism
P6	Male	10	Autism

P1 was a 10-year-old girl with autism. Her mother had canceled all her therapy sessions, since the beginning of the pandemic, so she did not get any other therapy for emotion recognition. Her mother reported that her main problem was in recognizing the fear emotion. She also had problems in her social interactions and did not know how to interact with others. For instance, she showed friendly behaviors with unfamiliar individuals and could not keep the right social distance, when speaking to others. In addition, she had stubborn behaviors and persisted in her requests without considering the situation.

P2 was a 6-year-old boy with autism who showed difficulty in recognizing sadness and fear emotions. His mother said that he did not enter other children’s group play and did not share his interests with others. He did not know the rules and norms of group play, so he was not successful in making good relationships with his peers. Moreover, he mostly expected confirmation of his actions from his parents, otherwise, he would become very nervous.

P3 was a 7-year-old boy with developmental delay and with major problems in recognizing anger and “fear” emotions. His mother complained that he was shy and could not speak and communicate comfortably with other people while he was good at doing his school homework. In addition, he had problems with the theory of mind ability and did not comprehend the concept of winning or losing in games or how to behave when he was a winner or a loser. It was also reported that he could not play properly with others.

P4 was also an 8-year-old boy who showed major difficulty in recognizing sad emotions. Also, his mother reported that he did not vocally express his emotions or express them on his face when he experienced emotional situations. In addition, he had major problems in his relationship with his classmates, because his social skills were lower than his peers. For example, he could not tolerate when he would lose in a game. Also, he rarely could pass theory of mind tests which was his major problem for which he was referred for further evaluations and interventions.

P5 was an 11-year-old boy with autism, whose major problem was recognizing anger and fear emotions. It was reported that he was addicted to playing computer games and did not attend social activities. In addition, he mostly wanted to leave in the middle of his therapy sessions. His therapist expressed that his language abilities were lower than his age group, so he rarely made a good connection with his peers. In addition, it was reported that he could have had better cognitive and social abilities if he had not left his therapy sessions periodically due to his good learning abilities.

P6, was a 10-year-old boy with autism diagnosis of severity level 2, with a major problem in recognizing anger and fear emotions. Also, he had difficulty recognizing happy facial expressions. Regarding expressing emotions, he could not show any emotions on his face. He had some movement difficulties and when he was left alone, he would start to tip-toe walking. His mother reported that he had low patience to wait when he demanded something. In addition, he rarely interacted with other people spontaneously and never initiated entering his peers’ games. Also, most of the time, he did not initiate communication unless to satisfy his needs. The others asked questions to communicate with him. Furthermore, he was behind his age in his language competencies compared to his age group.

It should be mentioned that none of the participants received other emotion recognition interventions during our study period.

### 3.3 Setting and materials

**FIGURE 3:**
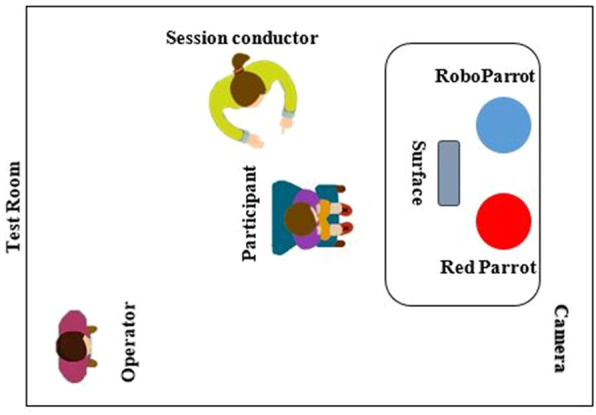
The overall schematic of the designed RSE.

The RSE setup, i.e., a 50 cm high table with two robots and a tablet, was set in a 3 × 3 meters room. The tablet was placed on a stand between the robots to display the images or run the designed games. The game was developed using the Unity game engine to show the desired emotions. A camera was positioned at one corner of the room to record the overall view of the setting so that we can analyze the sessions later. In each session, there was a psychologist who was overseeing the whole process to make sure everything run smoothly and according to general therapy rules. Furthermore, his feedback was collected for future improvements. Besides the therapist, the main researcher was present during all the sessions making sure that the sessions run according to the plan. The overall structure of the designed RSE can be seen in Figure 3.

### 3.4 Study design

To investigate the effect of the proposed RSE in teaching facial expressions (happiness, sadness, anger, and fear) to children with autism, a single case A-B-A (Cooper et al., 2014) design experiment with a generalization probe across novel stimuli and setting was conducted. The A-B-A technique is a reversal design that has three phases: 1) Phase A or the first baseline in which no treatment is introduced, and the behavior is evaluated, 2) Phase B in which the treatment is introduced, 3) Phase A (that we called it A′) or second baseline which is a return to the baseline by stopping the treatment. In this technique, the effectiveness of a proposed protocol is evaluated using multiple measures to see the changes by comparing Phase A to Phase B and then to Phase A’.

Within 1 week after baseline phase (A), a familiarization session was conducted to introduce the robots to the participants and reduce their novelty effect. In this session, the session conductor invited the participants to become familiar with the two robots. Then the robots started greeting the participants, introduced themselves, and asked the participants’ names, ages, and favorite activities to make a sense of friendship. Then the robots started playing with a robotic ball, called Sphero, to attract the participants (Soleiman et al., 2020). In this familiarization session, the participants were free to play with the robots. Within 1 week after this session, the training phase (B) was started.

#### 3.4.1 Phase A–First baseline

Before starting the training sessions, we evaluated the participants’ abilities in recognizing emotions in three sessions for 2 weeks. The images were selected from the Child Affective Facial Expression (CAFE) set (LoBue, 2014; LoBue & Thrasher, 2015; LoBue & Thrasher, 2018) and Radboud Faces Database (Langner, 2010) to have both adults’ and children’s images. Twenty images, 10 males and 10 females, were randomly selected from these datasets, including 5 images for every facial expression. The order of the images was also random to avoid memorizing the images based on the order. Each participant sat on a chair in front of a laptop computer and the session conductor presented the images one by one and asked the participant “what is her/his feeling?“, then she waited for 3s. If the participant did not look at the images, she delivered the question up to two additional times. No feedback was provided on the responses of the participants. At the end of the task, the participants were praised for their attentiveness. The participants scored one for each right answer and zero for each wrong answer or lack of an answer.

#### 3.4.2 Phase B–Therapy

**FIGURE 4:**
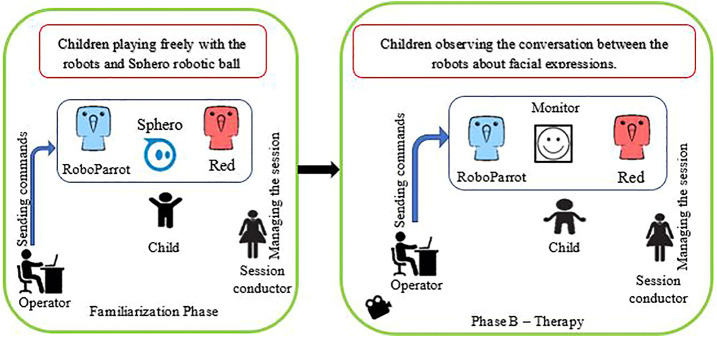
The Schematic of the Familiarization phase and Phase B Therapy.

The first therapy session was started 1 week after the familiarization session. A fixed protocol was given to the robots’ operator to run all the sessions. Thirty-two images of 4 emotions, i.e. 8 images per expression, were chosen to be displayed on the tablet. These images were different from the images that were used in the baseline phases to prevent children from memorizing them. The schematic of the familiarization and Phase B can be seen in Figure 4.

Before starting the therapy, the operator practiced the scenario several times. For all steps of the scenario, potential interaction sentences were determined. The scenario and the overall setup were tested by 5 members of our laboratory. They gave us feedback about the speed and ability of the operator to run the system. We used their comments to run the scenario smoother and more naturally. The steps of the scenario are as follows.

1. Robots greet the participant.
2. If the participant shows a tendency to speak to the robots, the robots answer him/her and give them feedback with laughing, movement, and funny voices.
3. The robots invite the participant to sit on a chair and watch them play.
4. The game is run for the robots to correctly name the emotions.
5. The robots start the conversation about the current image and name the emotion.
6. Another image is displayed, and this process repeats until the termination of the game (going back to step 5).
7. If the session ends, the robots say goodbye to the participant and invite him/her to the next session.

If there is any problem with the interaction that would cause any disruptive behavior the session conductor reacted accordingly.

**FIGURE 5:**
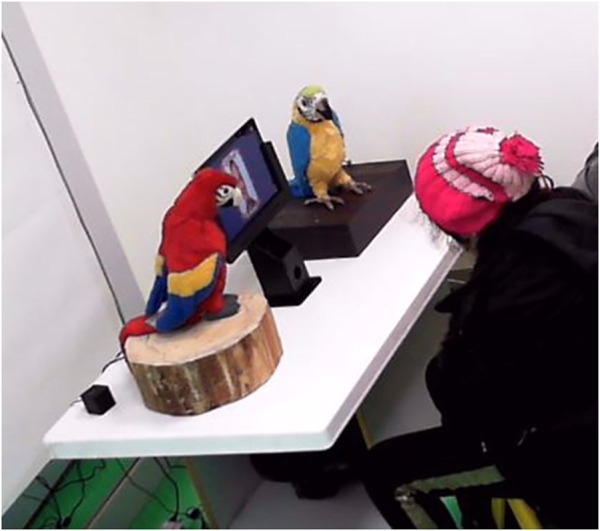
The placement of the robots and a subject.

In step 5, RoboParrot asked Red “what is this boy/girl/man/woman feeling?“, then Red answered the question and explained why that face convey that feeling, such as “he is laughing so he is happy”. Also, the robot may explain the same thing from the effect point of view of the emotion, i.e., “When we are happy, we may laugh”. As a verbal reward, Roboparrot praised Red saying “bravo” to reinforce his correct answer. Furthermore, a star or a flower was displayed on the screen as a visual reinforcement for Red. To ensure that the participants do not miss the named emotion, one of the robots would repeat the named emotion again. Every training session took a maximum of 30 min. A view of the experimental set-up is shown in Figure 5.

It should be mentioned that we had designed several questions and sentences based on our previous studies (Soleiman et al., 2020) such as “how are you? How old are you? sit on the chair, listen to me, what is your name? and I am fine”, to interact with children if they showed interest to interact. For instance, at the start of a conversation, P2 asked one of the robots “are you talking to your friend, is it possible to talk to me too?” or asked them “are you playing together”. Also, P4 hugged and touched them and asked their names. In such cases, the robots answered the participants. These behaviors mostly occurred at the beginning or end of the sessions.

At the end of each session, the participants were taken to another room and the researcher evaluated their emotion recognition capabilities, with a similar process to the baseline phase. Every participant had one or two training sessions weekly with at least 3 days gap, so the whole training phase took 8 weeks.

#### 3.4.3 Phase A'—Second baseline

Three sessions were conducted for the second baseline, which was similar to the first baseline. The second baseline was done 2 weeks after the training phase.

#### 3.4.4 Generalization probe

A generalization probe was conducted 2 weeks after the second baseline. In this session, a similar evaluation to the baselines was conducted with 20 new images that were randomly selected from Radboud and CAFE datasets. In other words, we wanted to show that the subjects did not only learn the emotions in the images used in the therapy sessions, but they learned to recognize the four emotions in a set of new images. This evaluation was designed and performed in a room different from the therapy room to see if children could generalize the newly-learned skills to unseen stimulus items and setting.

### 3.5 Evaluation and analysis

To evaluate the effects of the proposed RES on children’s emotion recognition skills, we used descriptive analysis, i.e., visualization of the gathered data from the sessions, and a non-parametric rank order correlation effect size measure, called TAU-U (Parker et al., 2011). This method helps interpret single-case experiments. Using graphs to show each participant’s data point in all sessions, enables us to track the level, trend, and changes within and between phases. Using these two methods together can make the evaluation more reliable (Brossart & Laid, 2018).

The TAU-U non-parametric analysis (Parker et al., 2011) is a useful and desirable method for single-case experiments to calculate and illustrate the effects of therapy on both within-phase trends and across-phase differences separately. It considers all pairwise comparisons of non-overlap points in a time-forward direction similar to the NAP method (non-overlap of all pairs). However, NAP is insensitive to the trend of the data and cannot consider the pre-existing trend of the baseline which would be an indicator of the participants’ improvement even without intervention. The pairwise comparisons in TAU-U result in three decisions of Pos (positive), Neg (negative), or Tie. Pos shows a score improvement and Neg shows a score decrement from Phase A to B. TAU-U is calculated based on the number of Pos and Neg pairwise points. It also considers controlling undesirable baseline monotonic trend with baseline correction by calculating Tau-U_A vs. B–trend A_ (Vannest et al., 2016) In this study three TAU-U calculations were considered: 1) Tau-U_trend A_ to control the trend of baseline, 2) Tau-U_A vs. B_ to evaluate the effect of the training, and 3) Tau-U_A vs. A’_ to compare the second baseline with the first baseline to examine the sustained effect of the training. When Tau-U_trend A_ ≥ 0.4, which means that there is a trend in the baseline, a baseline trend control is needed to remove its effect in the analysis process. The conditions for interpreting TAU-U are as follows: 1) above 0.8 is a very large change, 2) between 0.6 and 0.8 is a large change, 3) between 0.2 and 0.6 is a moderate change, and 4) 0.2 and below is considered a small change (Vannest & Ninci, 2015).

In addition to the above evaluation approaches, the mothers of participants were interviewed at the end of RSE therapy to see if they had observed any changes in their children’s abilities in recognizing and expressing emotions.

## 4 Results

### 4.1 Visual analysis

**FIGURE 6:**
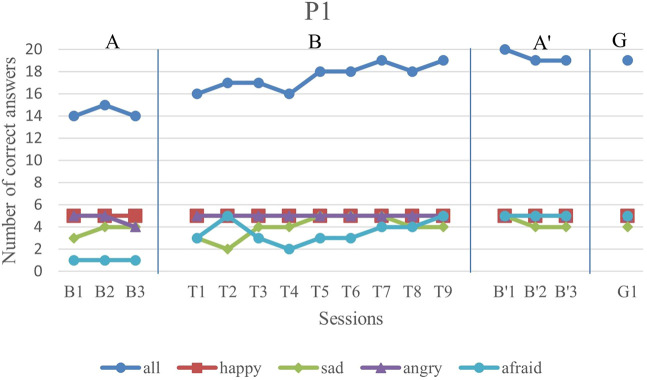
P1’s data points during all sessions—“all” refers to all correct answers of the participant.

**FIGURE 7:**
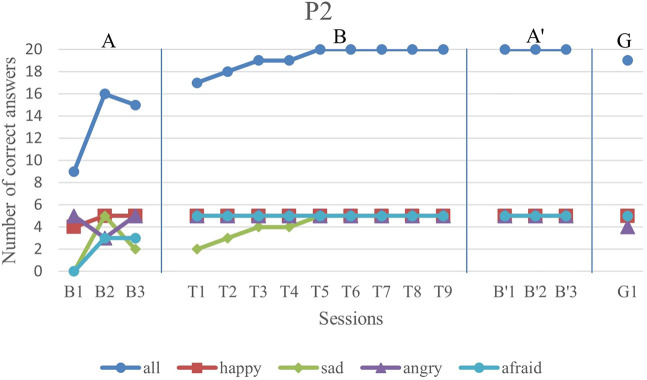
P2’s data points during all sessions - “all” refers to all correct answers of the participant.

**FIGURE 8:**
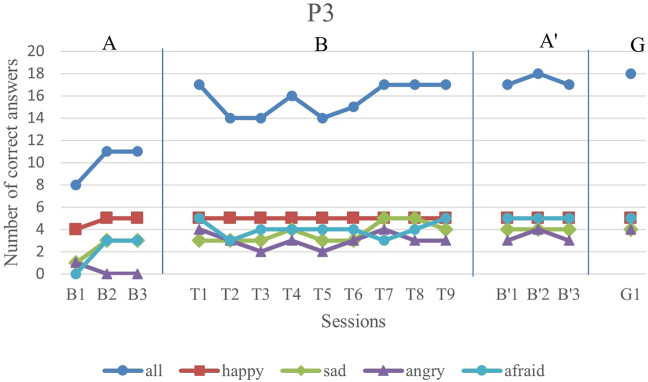
P3’s data points during all sessions - “all” refers to all correct answers of the participant.

**FIGURE 9:**
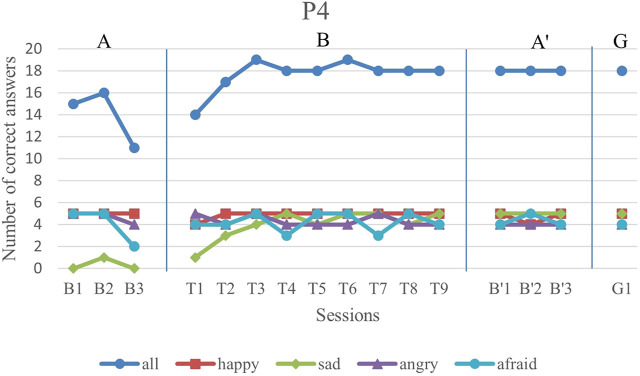
P4’s data points during all sessions - “all” refers to all correct answers of the participant.

**FIGURE 10:**
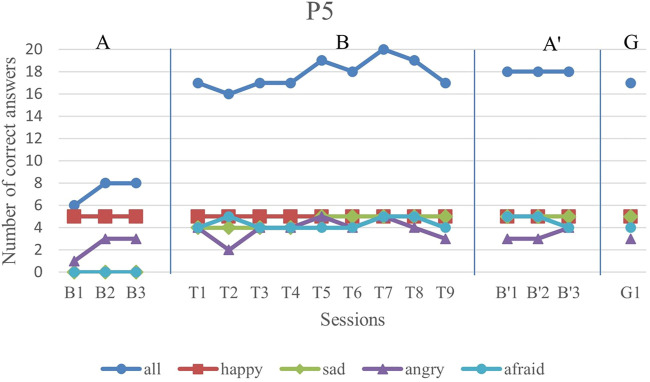
P5’s data points during all sessions - “all” refers to all correct answers of the participant.

**FIGURE 11:**
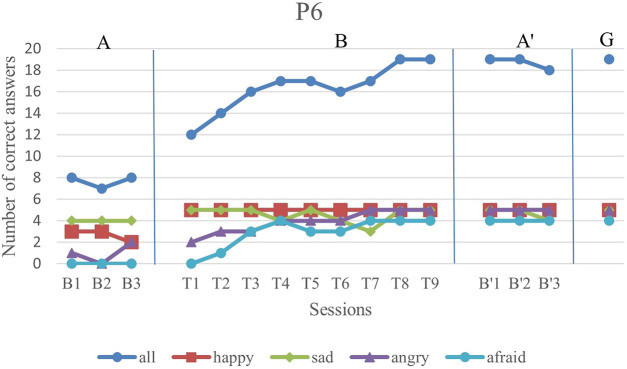
P6’s data points during all sessions - “all” refers to all correct answers of the participant.

We first analyze the results visually to see trends of data points in the study. Figures 6–11 show P1’s to P6’s correct number of responses during baseline (A), training (B), maintenance (A′), and generalization (G) probes. Visual analysis shows that in the first baseline phase, all participants had major problems or difficulty with one or two emotions.

P1’s major problem was with fear facial expression and her first baseline has a low steady trend with only one correct answer in each session. In the first and second training sessions, she exhibited good improvement. Although she showed a decrease in the fourth session, her trend line is gradually increasing for the sessions after it.

P2 did not have a major problem in any of the four emotions while he exhibited difficulty with fear and sadness emotions in the first baseline. His performance in both afraid and sad facial expressions had about 50% accuracy. After starting the training sessions, he could completely deliver correct answers for happiness, anger, and fear facial expressions. Furthermore, despite the variations in his sadness emotion recognition in the baseline, he showed a gradually increasing trend in this emotion.

P3, initially exhibited a major problem in anger emotion recognition, while he performed fairly well during the training sessions. Also, although he had difficulty with recognizing afraid and sad facial expressions, he had improvements in both emotions during the training sessions. The overall trend of his performance in all emotions was increasing, but with a low slope and several oscillations.

P4 exhibited a major problem with sad facial expression recognition with a steady trend in the first baseline. In his training sessions, the sad facial expression recognition had a gradually increasing trend with complete correct answers in the last training sessions.

P5 could not recognize any of the sadness and fear emotion images in the first baseline phase. But, as the training sessions began, he showed a considerable improvement in these two emotions. His overall trend line in all emotions was gradually increasing.

Finally, P6 exhibited major problems in anger and fear emotions with no correct answer for fear emotions during the baseline phase. After starting the intervention, the recognition of both of these emotions started to increase gradually. In terms of happiness emotion, he showed a complete performance in all intervention sessions. The only session in which he performed worse than his baseline in just sadness emotion was T7, in which he seemed less concentrated than in other sessions. However, in the rest of the sessions, his performance was increasing.

The overall performance, i.e., the blue drawing labeled as “all”, of all subjects follows an overall incremental trend in their training phase. In the case of P3, he showed a good improvement in the first session followed by oscillations, until it became stable at the end. The performance of the subjects at the end of each training session was higher than the first baseline sessions for all participants, except the first training session of P4. It should be noted that P5 expressed tiredness at the end of the last session, which may have resulted in his declining performance.

By evaluation of the second baseline, and the generalization probe, it can be concluded that all participants could maintain and generalize the learned skills. Furthermore, most of the performances of the participants in the individual emotions have become stable in the second evaluation and the follow-up. Specifically, P1, P2, P4, and P6 had over 90% correct answers and P3 and P5 had over 80% correct answers. The mean and standard deviation of the three phases for all participants’ overall scores and the generalization scores are shown in Table 2. As can be seen, the mean is greater in training and the second baseline phases than the first baseline for all participants.

**TABLE 2:** The mean and standard deviation of the three phases for all participants’ overall score and the generalization scores.

Participant	Phase A		Phase B		Phase A′		Phase G
	Mean	SD	Mean	SD	Mean	SD	
P1	14.33	0.47	17.56	1.07	19.33	0.47	19
P2	13.33	3.09	19.22	1.03	20	0	19
P3	10	1.41	15.67	1.33	17.33	0.47	18
P4	14	2.16	17.67	1.4	18	0	18
P5	7.3	0.94	17.78	1.22	18	0	17
P6	7.67	0.47	16.33	2.1	18.67	0.47	19

### 4.2 TAU-U analysis

We calculated Tau-U_trend A,_ Tau-U_A vs. B,_ and Tau-U_A vs. A’_ for all participants. As it was mentioned before, if there is a Tau-U_trend A_ ≥ 0.4, meaning there is a trend effect and it needs baseline control. Thus, the correction was done for P3 and P5. The results for comparison between the baseline and intervention as well as two baseline phases are shown in Table 3 (CI refers to Confidence Intervals). It indicates very large and large improvements for all children in both training and the A’ phase.

**TABLE 3:** TAU-U between phases values for all participants.

Participant	Label	TAU	*p*_Value	CI 85%	CI 90%
P1	Tau-U_A vs. B_	1	0.01	[0.423, 1]	[0.341, 1]
P1	Tau-U_A vs. A’_	1	0.04	[0.267, 1]	[0.162, 1]
P2	Tau-U_A vs. B_	1	0.01	[0.423, 1]	[0.341, 1]
P2	Tau-U_A vs. A’_	1	0.04	[0.267, 1]	[0.162, 1]
P3	Tau-U_A vs. B_	0.92	0.02	[0.349, 1]	[0.267, 1]
P3	Tau-U_A vs. A’_	0.78	0.12	[0.045, 1]	[-0.060, 1]
P4	Tau-U_A vs. B_	0.85	0.03	[0.275, 1]	[0.193, 1]
P4	Tau-U_A vs. A’_	1	0.04	[0.267, 1]	[0.162, 1]
P5	Tau-U_A vs. B_	0.92	0.02	[0.349, 1]	[0.267, 1]
P5	Tau-U_A vs. A’_	0.78	0.12	[0.045, 1]	[-0.060, 1]
P6	Tau-U_A vs. B_	1	0.01	[0.423, 1]	[0.341, 1]
P6	Tau-U_A vs. A’_	1	0.04	[0.267, 1]	[0.162, 1]

In addition to calculating Tau-U for every individual, the weighted average Tau-U was used to compare the first baseline and the intervention as well as between the first baseline and the second baseline. The combination of contrasts of all participants as well as Variances (Var-Tau), Z scores (Z), *p* values (*p*-value), and Confidence Intervals (CI) are shown in Table 4. As can be seen the Tau-U for both comparisons show a very large improvement in emotion recognition in the training and the second baseline with *p* < 0.01.

**TABLE 4:** Weighted TAU-U between phases values for all participants.

Label	Tau	Var-Tau	Z	*p*-Value	CI 85%	CI 90%	CI 95%
Tau-U_A vs. B_	0.95	0.16	5.81	<0.001	[0.715, 1]	[0.682, 1]	[0.630, 1]
Tau-U_A vs. A’_	0.93	0.20	4.45	<0.001	[0.627, 1]	[0.584, 1]	[0.519, 1]

### 4.3 The mothers’ interview results

When the therapy period ended, an approximately 10–15-min interview with the participants’ mothers was conducted. They were invited to a quiet place in the autism center and were asked to describe the changes that they had recognized in their children’s emotion recognition as well as emotion expression skills. The interview questions were: 1) Have you noticed any changes in your child’s emotional expression ability after the RSE-based therapy? 2) Have you noticed any changes in your child’s emotion recognition ability after the RSE-based therapy? 3) Describe or give examples of your child’s new reactions or expressions in emotional situations after the RSE-based therapy? In the following, the summary of the interviews with the participants’ mothers is reported.

**P1:** Her mother reported that she rarely spoke about her own or others’ emotions spontaneously and could not recognize fear emotion before entering the therapy sessions. However, after the RSE therapy sessions when she was watching cartoons and movies, she started speaking about characters’ emotions with sentences such as “this man is afraid”. In addition, when the family members expressed their emotions, she could recognize it, and sometimes spoke/asked about it, e.g., she asked “mom, are you angry?“. Also, her mother noticed that not only she expressed her emotions better than before, but also, she spoke about her own emotions more with sentences such as “mom, my brother scared me.”

**P2:** His mother reported that he became very active and reacted to happy situations more than before. He also showed happy emotions and feelings more than before.

**P3:** His mother reported no noticeable changes in his emotions.

**P4:** His mother reported that he had never expressed his feeling, facially or verbally. Furthermore, he had never spoken about other family members’ feelings and emotions before entering the RSE-based therapy sessions. However, after the therapy sessions, he started to have facial expressions of his emotions and spoke about them, with sentences such as “I am happy now”. In addition, he started speaking about others’ feelings with sentences such as “Dad is angry”.

**P5:** His mother reported that she noticed that her son reacted to his parents’ emotions more than before and asked, “what happened?” when they were angry or sad. Furthermore, he reacted differently to scary scenes in the movies and preferred not to watch such scenes and asked her mother “are you afraid?“.

**P6:** His mother reported that after the RSE-based therapy sessions, he could recognize the emotions of characters in movies and cartoons which did not happen before. She expressed that his son had been indifferent to their facial expression before the therapy sessions but became more sensitive about their emotions after the therapy sessions.

## 5 Discussion and conclusion

In this study, we investigated the idea of using RSE for teaching emotion recognition through observational learning to children with autism. This study validated the previous work (Soleiman et al., 2020) which showed that RSE can be helpful in teaching skills to children with autism. In the current design of using RSE, children observed two parrot-like robots labeling 4 basic emotions and explaining the characteristic and emotional cues in a picture of a face. It should be noted that we used different sets of images in the baselines and the intervention phases to prevent children from pure memorization. We used animal-based RSE rather than humanoid-based RSE since based on Ricks and Colton’s study (2010), non-humanoid robots usually elicit a better engagement on tasks.

The results showed that children can learn to recognize four emotions by observing this fully robotic social environment with no direct human intervention. The visual analysis revealed that all children improved their emotion recognition skills and showed a stable performance at the end of the training sessions. In addition, to evaluate the effectiveness of the proposed therapy, we used the TAU-U method which is helpful for descriptive and inferential analysis in single case design. All TAU-U values were over 0.7 for all children in the training phase which revealed a very large and large effect. Furthermore, the comparison of the maintenance and the generalization probes with the first baseline showed that the effect of the training remained even after the therapy sessions were over.

The findings of this study strengthen the hypothesis that RSE can be a useful replacement or complement for therapeutical social environments. This replacement becomes especially important when it is hard to provide or benefit from a human social environment for children with autism. Furthermore, the interview with the participants’ mothers revealed that their children did not use to participate in group plays and avoided being in social environments. In contrast, the children accepted our RSE, stayed until the end of the sessions, and tried to speak to the robots. Furthermore, the mothers’ interviews showed that most of the participants could learn and generalized the therapy results to their actual life.

Our study results are consistent with the results of the previous studies (Saadatzi et al., 2018; Ali et al., 2019; So et al., 2020) that have shown the effectiveness of a therapy or training environment without human involvement in autism therapy. However, the work by Saadatzi et al. (2018) simulates a classroom environment in which an avatar teaches a robot and a child with autism. Thus, it is limited to a classroom setup. On the other hand, the work by Ali et al. (2019), in which two Nao robots are used to teach joint attention and imitation, does not benefit from social interaction and simply uses different stimuli for intervention. Finally, the closest work to our work is by So et al. (2020) which has shown that drama play could affect the joint attention and functional play of children with autism.

In this study, we wanted to show the effect of a full RSE focusing on social skills. In our study, we tried to simulate a real conversation between two robots that lasted during the whole session to attract children to their conversation, which was based on the skill that we wanted to teach. It should be noted that although our robots did not have facial expressions, the children could learn from their interaction.

Finally, the importance of using an RSE and its extra complexity and cost, compared to single robot environments, is in its capability to provide observational learning and multi-party social interaction through interaction between several robots.

It should be noted that our study had several limitations: 1) the study was run on a small number of participants in a single-subject format with no control group, 2) the robots had no active faces and could not show emotions, which could make the session more realistic, 3) limited capabilities of the robots for pointing to different parts of the faces to attract children’s attention during their explanation about facial cues, 4) the maintenance check was not performed long after the therapy time. Despite these limitations, the fact that the robots labeled the emotions and explained their signs and characteristics of them made it unique in this observational learning for the participants.

For our future research, we are designing a robot with facial expressions to see if it is more attractive for children. In addition, we want to investigate the effect of RSE on more children with different levels of autism with a control group with more skills. The autonomy and mobility of the robots are two other factors that are under revision for future work on the proposed RSE.

In the end, we like to point out an important area of research and concern regarding the ethical issues related to the use of social robots. There are several criticisms (Turkle, 2011; Lin et al., 2012) point to the illusion of affective relation between robots and humans, which may negatively affect vulnerable users such as children and elderlies. The argument is that this illusion, especially when it comes to participants with special needs, results in social isolation. On the other hand, some researchers believe that future robots would be advanced enough to provide realistic affective relations with humans (Danaher, 2019). Furthermore, some researchers believe that the benefits of social robots can be employed while considering ethical issues (Damiano, & Dumouchel, 2018; 2020). Although our study did not directly address these ethical issues, it introduces new benefits of social robots that should be considered in the discussions over ethical issues of using social robots. 

## Data Availability

The original contributions presented in the study are included in the article/supplementary material, further inquiries can be directed to the corresponding author.
